# Validity and Reliability of the Burnout Assessment Tool (BAT) in Serbian

**DOI:** 10.3390/healthcare14030317

**Published:** 2026-01-27

**Authors:** Zorica Terzic-Supic, Konstantinos Stratakis, Teresa Candido, Zorana Nikolov, Milivoje Galjak, Dejan Nesic, Goran Aleksandric, Dejan Radaljac, Jovana Todorovic

**Affiliations:** 1 Faculty of Medicine, Institute of Social Medicine, University of Belgrade, Dr Subotica 15, 11000 Belgrade, Serbia; 2 Faculty of Medicine, University of Belgrade, Dr Subotica 8, 11000 Belgrade, Serbia; kostasstrata7@gmail.com (K.S.); teresacandido55@gmail.com (T.C.);; 3 Faculty of Medicine, University of Pristina, 38220 Kosovska Mitrovica, Serbia; 4 Faculty of Medicine, Institute of Medical Physiology, University of Belgrade, Visegradska 26, 11000 Belgrade, Serbia; 5 Clinical Hospital Center Zemun, Vukova 9, 11080 Belgrade, Serbia

**Keywords:** burnout, BAT, burnout assessment tool, validity

## Abstract

**Background**: Burnout is a syndrome resulting from long-term, unmanaged work-related stress. This study aimed to evaluate the validity and reliability of the Serbian versions of BAT among fifth-year medical students at the University of Belgrade Faculty of Medicine. **Methods**: This cross-sectional study, which included a total of 431 students at the Faculty of Medicine, University of Belgrade, was conducted during the last week of November 2024. The study instruments used were the Burnout Assessment Tool (BAT) and the Copenhagen Burnout Inventory (CBI). **Results**: Cronbach’s alpha for the entire BAT scale was α = 0.946; for core burnout symptoms, it was α = 0.938; for the exhaustion scale, α = 0.865; for mental distance, α = 0.858; for cognitive impairment, α = 0.907; for emotional impairment, α = 0.878; for secondary symptoms, α = 0.863; for psychological distress, α = 0.791; and for psychosomatic complaints, α = 0.801. The EFA showed six factors that explained a total of 63.76% of the variance. Factor 1 explained 35.71% of the variance; factor 2 explained 9.81%; factor 3, 5.785%; factor 4, 5.415%; factor 5, 3.956%; and factor 6 explained 3.076% of the variance. After the elimination of the three items with the lowest loadings, the EFA showed five factors that explained a total of 63.347% of the total variance. Factor 1 explained a total of 36.637% of the variance; factor 2, 10.544%; factor 3, 6.345%; factor 4, 5.612%; and factor 5 explained a total of 4.209%. **Conclusions**: This study showed that the Serbian version of the BAT exhibits excellent reliability, clear factorial validity, and strong convergent and discriminative performance.

## 1. Introduction

The International Classification of Diseases, 11th Revision (ICD-11), defines burnout as a syndrome that results from long-term, unmanaged work-related stress [1]. It involves feelings of exhaustion, mental detachment or cynicism, and a lower sense of effectiveness, all of which negatively impact both physical and mental health and performance [1]. Burnout is not typically viewed as a medical condition; instead, it is considered an occupational issue that arises from how individuals interact with their work environment [2]. This view emphasizes that burnout is a dynamic process influenced by external demands and available resources, rather than being solely based on fixed personal traits [2].

Burnout is a growing concern in higher education [3]. University students often move to new cities, face academic challenges, gain financial independence, initiate romantic relationships, and assume community responsibilities, all while being separated from the support systems usually provided by friends and family [3]. These rapid and significant changes make them more vulnerable to burnout [3]. Medical students are at an even higher risk due to the unique challenges they face during their training [4]. These challenges include early clinical responsibilities, exposure to death and suffering, and, in particular, the workload and the long duration of their studies [4]. As a result, almost 50% of medical students develop symptoms of burnout at some point during their studies [5]. This can lead to various adverse effects, such as mental health issues, lower cognitive performance, and increased dropout rates [6,7]. Thus, early detection and ongoing monitoring of burnout are crucial to prevent these adverse outcomes [8].

Measuring burnout accurately is crucial for developing effective strategies to prevent and manage it. The Copenhagen Burnout Inventory (CBI) was designed to enhance the understanding of burnout by examining both emotional and physical exhaustion [9]. It assesses burnout in three areas: personal burnout, work-related burnout, and client or colleague-related burnout [9]. Its flexibility has made it valuable across different groups, including students [9]. The Burnout Assessment Tool (BAT) is a newer measure that captures modern theoretical insights. Unlike older tools, the BAT sees burnout as a syndrome made up of four main dimensions—exhaustion, mental distance, cognitive impairment, and emotional impairment [10]. It also considers secondary symptoms such as psychological distress and physical complaints [10]. By incorporating both primary and secondary symptoms, the Burnout Assessment Tool (BAT) provides a complete view of how burnout impacts individuals [11].

Previous research in the Serbian population relied on the Maslach Burnout Inventory (MBI), the Study Burnout Inventory (SBI), and the CBI to identify burnout syndrome [12,13]. Although international studies have confirmed the reliability and validity of the BAT, no research has focused on the Serbian population, especially among medical students. This represents a significant gap, as instruments that are not psychometrically validated in a specific language may fail to accurately capture the actual prevalence or nature of burnout, thereby limiting both research comparability and the design of effective preventive programs [14]. Therefore, this study aimed to evaluate the validity and reliability of the Serbian versions of BAT among fifth-year medical students at the Faculty of Medicine, University of Belgrade.

## 2. Materials and Methods

This cross-sectional study included a total of 431 students at the Faculty of Medicine, University of Belgrade, and was conducted during the last week of November 2024. The participants were fifth-year medical students who were attending classes in Social Medicine during the study week. All students present at the classes during the week of the study were eligible for participation. All participants were given information about the study, its processes, and aims, and they gave consent for participation. The Ethical Committee of the Faculty of Medicine, University of Belgrade, approved the study (No. 25/IX-4, 16 September 2024). The participants were then asked to fill in and return the questionnaire, which was printed on paper. The response rate was 100%.

The study instruments used were the Burnout Assessment Tool (BAT) [10] and the Copenhagen Burnout Inventory (CBI) [9]. The Burnout Assessment Tool is a 33-item tool that measures burnout in four main dimensions—exhaustion, mental distancing, cognitive impairment, and emotional impairment—and two secondary dimensions: psychological stress and psychosomatic complaints [10]. The four main dimensions represent core symptoms of burnout. The answers on the BAT are given on a 5-point Likert scale, ranging from 1 (never) to 5 (always). The total score on the BAT questionnaire is calculated as the mean of all answers on the scale, and the score on each dimension is presented as the mean score on that dimension [10]. The Burnout Assessment Tool was translated to Serbian through forward and then backward translation, all in accordance with the recommendations of the World Health Organization [15]. Pilot testing was then performed in order to determine whether the questions were clear and understandable and whether their order was correct, as well as to examine the time that was required for the completion of the questionnaire.

The Copenhagen Burnout Inventory (CBI) measures burnout in three domains, personal burnout, work-related burnout, and client-related burnout [9], provided on a five-point Likert scale ranging from 0 (never) to 4 (always). The version for students validated in Serbian [12] has the following domains: personal burnout, studying-related burnout, and faculty member-related burnout. For the total score on the scale, each of the answers to each of the items is transformed into percentages: 0 (never) = 0; 1 (rare) = 25%; 2 (sometimes) = 50%; 3 (often) = 75%; and 4 (always) = 100%. The score on each domain is calculated as an average percentage for that domain, and the total score on the scale is calculated as an average score of the three domains [9,12].

Statistical analyses were performed using descriptive and analytical statistics. Cronbach’s alpha was used to assess the internal consistency of the BAT questionnaire. Exploratory factor analysis (EFA) was used for the exploration of the factor structure, and the extraction of the factors was conducted using the Promax rotation with the assumption that the factors are correlated. Suitability of data for factorisation was examined using the Kaiser–Meyer–Olkin (KMO) measure of sampling adequacy and Bartlett’s test for sphericity. The preferable result of Barltett’s test was significant. The criterion for loading was set at 0.6, and all items with loading below 0.6 were deleted [16]. The number of factors was determined based on the results of the exploratory factor analysis (EFA) conducted in SPSS. Specifically, we relied on eigenvalues greater than 1 (Kaiser criterion), which indicated the presence of six factors in the initial solution. After the elimination of the items with the lowest loadings, we conducted the EFA once more, and it showed the presence of five factors with eigenvalues above 1. The exploratory factor analysis was performed on a random 50% of the sample. Confirmatory factor analysis was performed on a second 50% of the sample. There were no missing data on the items of BAT, and conditions for conduction of CFA were fulfilled. Goodness-of-fit indices used were Root Mean Square Error of approximation (RMSEA ≤ 0.07) and comparative fit index (CFI > 0.95). The statistical analyses were performed using the Statistical Package for Social Sciences (SPSS) 22.0 and Advanced Mortar System (AMOS) 22.0 (Armonk, NY, USA).

## 3. Results

The study included total of 431 participants; 128 were male (29.7%), 302 were female (70.1%), and 1 participant did not report their sex (0.1%). The mean age of the participants was 23.52 ± 1.26.

The mean score on the Copenhagen Burnout Inventory was 42.09 ± 15.61. The mean score on the personal burnout scale was 42.87 ± 19.93; on study-related burnout, it was 46.03 ± 17.14; on colleague-related burnout, 37.56 ± 20.84; and on faculty member-related burnout, it was 41.90 ± 21.95. The prevalence of total burnout according to the CBI was 29.9% (129/431); prevalence of personal burnout was 35% (151/431); prevalence of study-related burnout was 36.9% (159/431); and prevalence of colleague-related burnout was 19.7% (85/431), while the prevalence of faculty member-related burnout was 29% (125/431).

The mean score on the BAT was 2.27 ± 0.64; on the exhaustion subscale, it was 2.87 ± 0.72; on the mental distance subscale, it was 1.96 ± 0.80; on the cognitive impairment subscale, it was 2.12 ± 0.84; on the emotional impairment subscale, it was 2.16 ± 0.87; on the psychological distress subscale, it was 2.52 ± 0.84; on psychosomatic complaints, it was 2.01 ± 0.81; and on the secondary symptoms subscale, it was 2.27 ± 0.75.

The 25th percentile for the total BAT score was 1.78; the 75th was 2.69; and the 95th was 3.41. The percentiles for the cutoffs of the BAT scale are presented in Table 1.

The prevalence of low burnout on the BAT scale was 25.4%, the prevalence of average burnout was 49.6%, the prevalence of high burnout was 20%, and the prevalence of very high burnout was 4.9%.

Cronbach’s alpha for the entire BAT scale was α = 0.946; for core burnout symptoms, it was α = 0.938; for the exhaustion scale, it was α = 0.865; for mental distance, it was α = 0.858; for cognitive impairment, it was α = 0.907; for emotional impairment, it was α = 0.878; for secondary symptoms, α = 0.863; for psychological distress, α = 0.791; and for psychosomatic complaints, α = 0.801.

The EFA showed six factors that explained a total of 63.76% of the variance. Factor 1 explained 35.71% of the variance; factor 2, 9.81%; factor 3, 5.785%. Factor 4 explained 5.415%; factor 5 explained 3.956%; and factor 6 explained 3.076% of the variance. The KMO measure of sampling adequacy was 0.915, and Bartlett’s test of sphericity was *p* < 0.01. The factor loadings varied from 0.487 to 0.885. Exploratory factor analysis with promax rotation and Kaiser normalization is presented in Table 2.

After the elimination of three items with the lowest loadings, the EFA showed five factors that explained a total of 63.347% of the total variance. Factor 1 explained a total of 36.637% of the variance; factor 2, 10.544%; factor 3, 6.345%; factor 4, 5.612%; and factor 5 explained a total of 4.209%. KMO = 0.913, and Bartlett’s test of sphericity was *p* < 0.001. The factor loadings in the optimized model varied from 0.606 to 0.875. Cronbach’s alpha for the entire BAT scale with eliminated items was α = 0.942; for core burnout symptoms, it was α = 0.939; for exhaustion, α = 0.861; for mental distance, α = 0.858; for the cognitive impairment scale, α = 0.903; for emotional impairment, α = 0.894; and for psychosomatic complaints, α = 0.815. Exploratory factor analysis with Promax rotation and Kaiser normalization of the optimized model is presented in Table 3.

**Figure 1:**
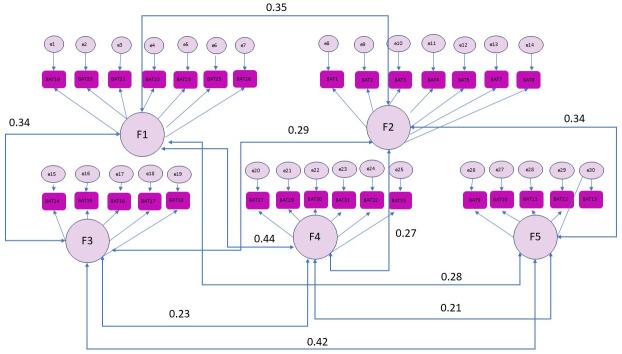
CFA.

Confirmatory factor analysis (CFA) for the five-factor model showed CFI 0.877 and RMSEA 0.07 (95% CI: 0.066–0.079). The results of the CFA are presented in Figure 1.

**Figure 2:**
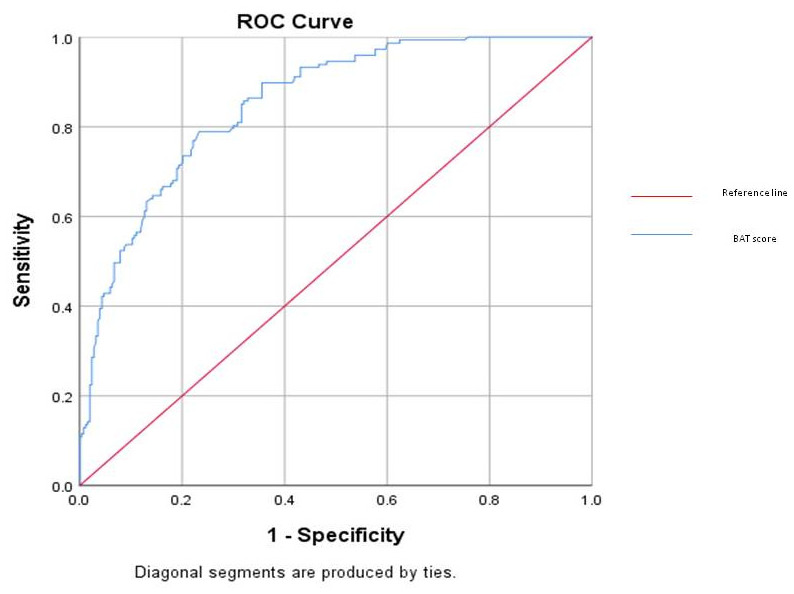
ROC curve.

The correlation between the BAT total score and CBI total score was 0.732 *p* < 0.001. Sensitivity of the BAT score for predicting burnout according to the CBI for a score of 2.20 (the 50th percentile of BAT score) was 0.823, and specificity was 0.684. Sensitivity for the 75th percentile score of 2.69 was 0.537, and specificity was 0.909. Total area under the curve was 0.853. The ROC curve is presented in Figure 2.

## 4. Discussion

The aim of this study was to evaluate the validity and reliability of the Serbian versions of the BAT among fifth-year medical students at the Faculty of Medicine, University of Belgrade, Serbia.

The BAT exhibited outstanding internal consistency, with Cronbach’s alpha values exceeding 0.94 for the full scale and consistently above 0.79 for its subscales. These findings align with the original Flemish validation, which also reported adequate reliability across all primary and secondary dimensions of the questionnaire [10]. Moreover, our results are in accordance with a recent meta-analysis confirming a similarly high reliability (pooled a = 0.798–0.948) across varied populations and BAT versions [17]. This evidence supports the instrument’s reliability in measuring burnout as a unified concept, which outperforms older tools like the Maslach Burnout Inventory (MBI), where some subscales, such as depersonalization and personal accomplishment, show lower internal consistency [18]. However, the overall Cronbach’s alpha may indicate redundancy of items [19], and further studies on BAT should tackle this as well. In our study, Cronbach’s alpha for the entire BAT was 0.946 for the original scale and 0.942 for the scale with removed items, which is completely in line with the results of previous studies, as shown in the mentioned meta-analysis [17].

Factor analysis showed high construct validity for the BAT questionnaire. The initial six-factor solution explained 63.76% of the variance. After removing the three items with the lowest factor loadings, a five-factor model emerged. This updated model included the factors of emotional impairment, exhaustion, cognitive impairment, psychosomatic complaints, and mental distance, with factor loadings ranging from 0.606 to 0.875, accounting for 63.347% of the total variance.

The factor loadings observed in our study may reflect cultural and contextual influences specific to medical students in our setting. The item with the lowest loading was ‘I have trouble falling or staying asleep’. Many medical students may feel that there is a need for studying all night and that there is respect to be gained from not sleeping and therefore do not perceive this as difficulty. This item is accompanied by two items, ‘I want to be active during my studies, but somehow I am unable to manage’ and ‘Crowds and noise disturb me’, both of which also refer to what can almost be described as a norm in the context of medical students. After the elimination of items with the lowest loadings, the secondary symptoms were clustered in one factor only, showing that mental and physical symptoms of burnout tend to coexist. Confirmatory factor analysis (CFA) showed an acceptable fit along with a subthreshold for comparative fit index (CFI = 0.877) and RMSEA of 0.07, indicating a less than ideal but somewhat acceptable structure. It is important to note that goodness-of-fit cutoffs are guidelines rather than strict rules, and several authors caution against rigid application of the 0.90 threshold, especially in complex models or with ordinal data. Moreover, given that this was the first validation of the questionnaire in Serbian, slight deviations from ideal fit indices are not unexpected. Our study’s results are aligned with evidence confirming the BAT’s multidimensional structure [18]. In the original Flemish validation, Schaufeli et al. (2020) identified four interrelated dimensions [10], while studies in Romania and Chile confirmed five- or six-factor models [20,21]. These findings emphasize the BAT’s robust and adaptable factorial structure across various contexts.

Our findings indicate that BAT scores demonstrate a strong correlation with scores from the CBI, thereby confirming the convergent validity of the BAT questionnaire for assessing burnout. This aligns with earlier studies that have established a robust correlation between the BAT and other recognized instruments for identifying burnout, such as the MBI and the Oldenburg Burnout Inventory (OLBI) [10,22]. Receiver operating characteristic (ROC) analyses yielded an area under the curve (AUC) of 0.853, suggesting an acceptable discriminative power of the BAT in identifying individuals experiencing burnout. At a cutoff score of 2.20 (median), sensitivity was high (0.823), though specificity was moderate but acceptable (0.684), whereas the higher cutoff of 2.69 improved specificity (0.909) but reduced sensitivity (0.537). This trade-off highlights the importance of context, with lower cutoffs being more suitable for screening, while higher cutoffs may be more useful for confirming burnout in clinical or occupational health settings [22]. Given the high sensitivity and acceptable specificity of the 2.20 cutoff, it is the proposed cutoff for the identification of burnout in the population of medical students in Serbia. However, it is important to note that in the examination of discriminative validity, the score on the CBI was used as the gold standard. A score indicative of burnout on the CBI does not equal clinically assessed burnout, and the BAT is discriminative compared to the other self-reported measure, the CBI.

Overall, our study shows that the BAT is a reliable and valid measure of burnout in higher education. It can identify different symptom areas, allowing institutions to create tailored support strategies, such as cognitive skill training, emotional counseling, and curriculum changes. For example, universities could implement initiatives like workload moderation, resilience programs, faculty teaching support, and accessible mental health services. By focusing on individual coping strategies and systemic stressors, these interventions are more likely to lower burnout rates and improve student well-being.

A key strength of this study is its thorough psychometric evaluation of the Serbian version of the Burnout Assessment Tool (BAT). This evaluation includes internal consistency, exploratory and confirmatory factor analyses, convergent validity, and discriminative validity compared to the Copenhagen Burnout Inventory (CBI). The relatively large sample of medical students from multiple institutions also boosts the generalizability of the findings within the Serbian academic setting. However, the sample includes only fifth-year medical students. While this is relevant to a high-risk group, it limits the generalizability to other student populations or healthcare professionals. Another limitation is in the cutoff we used for the factor detention and item deletion, as the fixed loading was set at 0.60.

## 5. Conclusions

Assessing burnout and its associated factors is essential for developing programs that enhance well-being and improve mental health and productivity. However, effective assessment requires reliable tools that are psychometrically validated in a specific language. This study showed that the Serbian version of the BAT exhibits excellent internal consistency, acceptable factorial validity, and convergent and discriminative performance in a sample of fifth-year medical students from a single Serbian medical faculty. These findings suggest that the BAT can be appropriately used to assess burnout within this specific population. Nevertheless, further studies involving medical students from other years, other institutions, and broader samples are needed to confirm the applicability and generalizability of the BAT across the wider Serbian population.

## Figures and Tables

**Table 1:** The 25th, 75th, and 95th percentiles on the BAT scale and its subscales.

Subscale	25th Percentile	75th Percentile	95th Percentile
Total BAT score	1.78	2.69	3.41
Core symptoms	1.76	2.70	3.44
Secondary symptoms	1.70	2.70	3.66
Exhaustion	2.37	3.37	4.00
Mental distance	1.40	2.40	3.60
Cognitive impairment	1.40	2.60	3.72
Emotional impairment	1.40	2.00	3.80
Psychological distress	1.80	3.20	4.00
Psychosomatic complaints	1.40	2.40	3.52

**Table 2:** Exploratory factor analysis with promax rotation and Kaiser normalization.

Item	Factor 1	Factor 2	Factor 3	Factor 4	Factor 5	Factor 6
Due to my studies, I feel mentally exhausted			0.680			
Everything I do for my studies requires a great deal of effort						0.816
After a day working on my study, I find it hard to recover my energy			0.775			
While working on my studies, I feel physically exhausted			0.777			
When I get up in the morning, I lack the energy to get started with my studies			0.660			
I want to be active when I am working on my studies, but somehow I am unable to manage			0.585			
When I exert myself for my studies, I quickly get tired			0.817			
At the end of day of working on my studies, I feel mentally exhausted and drained			0.790			
I struggle to find any enthusiasm for my studies					0.718	
When I am working on my studies, I do not think much about what I am doing and I function on autopilot					0.629	
I feel a strong aversion towards my studies					0.788	
I feel indifferent about my studies					0.885	
I’m cynical about the importance of my studies					0.836	
When I am working on my studies, I have trouble staying focused		0.766				
When I am working on my studies I struggle to think clearly		0.828				
I’m forgetful and distracted when I am working on my studies		0.836				
When I’m working on my studies, I have trouble concentrating		0.868				
I make mistakes while working on my studies because I have my mind on other things		0.798				
I feel unable to control my emotions	0.821					
I do not recognize myself in the way I react emotionally	0.732					
I become irritable when things don’t go my way	0.829					
I get upset or sad without knowing why	0.860					
I may overreact unintentionally	0.787					
I have trouble falling or staying asleep				0.487		
I tend to worry	0.713					
I feel tense and stressed	0.737					
I feel anxious and/or suffer from panic attacks				0.693		
Noise and crowds disturb me				0.589		
I suffer from palpitations or chest pain				0.755		
I suffer from stomach and/or intestinal complaints				0.749		
I suffer from headaches				0.761		
I suffer from muscle pain, for example in the neck, shoulder or back				0.755		
I often get sick				0.609		

**Table 3:** Exploratory factor analysis with Promax rotation and Kaiser normalization of the optimized model.

Item	Factor 1-Emotional Impairment	Factor 2- Exhaustion	Factor 3-Cognitive Impairment	Factor 4-Psychosomatic Complaints	Factor 5-Mental Distance
Due to my studies, I feel mentally exhausted		0.797			
Everything I do for my studies requires a great deal of effort		0.606			
After a day working on my study, I find it hard to recover my energy		0.795			
While working on my studies, I feel physically exhausted		0.851			
When I get up in the morning, I lack the energy to get started with my studies		0.645			
When I exert myself for my studies, I quickly get tired		0.723			
At the end of day of working on my studies, I feel mentally exhausted and drained		0.756			
I struggle to find any enthusiasm for my studies					0.739
When I am working on my studies, I do not think much about what I am doing and I function on autopilot					0.634
I feel a strong aversion towards my studies					0.804
I feel indifferent about my studies					0.875
I’m cynical about the importance of my studies					0.824
When I am working on my studies, I have trouble staying focused			0.744		
When I am working on my studies I struggle to think clearly			0.827		
I’m forgetful and distracted when I am working on my studies			0.853		
When I’m working on my studies, I have trouble concentrating			0.873		
I make mistakes while working on my studies because I have my mind on other things			0.813		
I feel unable to control my emotions	0.822				
I do not recognize myself in the way I react emotionally	0.734				
I become irritable when things don’t go my way	0.830				
I get upset or sad without knowing why	0.857				
I may overreact unintentionally	0.781				
I tend to worry	0.710				
I feel tense and stressed	0.736				
I feel anxious and/or suffer from panic attacks				0.678	
I suffer from palpitations or chest pain				0.730	
I suffer from stomach and/or intestinal complaints				0.751	
I suffer from headaches				0.755	
I suffer from muscle pain, for example in the neck, shoulder or back				0.760	
I often get sick				0.658	

## Data Availability

The raw data supporting the conclusions of this article will be made available by the authors on request.
